# Effect of Empagliflozin on Sphingolipid Catabolism in Diabetic and Hypertensive Rats

**DOI:** 10.3390/ijms23052883

**Published:** 2022-03-07

**Authors:** Roxana Pérez-Villavicencio, Javier Flores-Estrada, Martha Franco, Bruno Escalante, Oscar Pérez-Méndez, Adriana Mercado, Rocio Bautista-Pérez

**Affiliations:** 1 Department of Molecular Biology, Instituto Nacional de Cardiología “Ignacio Chávez”, Mexico City 14080, Mexico; rox.pv.27@gmail.com (R.P.-V.); opmendez@yahoo.com (O.P.-M.); 2 División de Investigación, Hospital Juárez de México, Mexico City 07760, Mexico; jose.florese@salud.gob.mx; 3 Department of Physiology, Instituto Nacional de Cardiología “Ignacio Chávez”, Mexico City 14080, Mexico; 4 Department of Cardio-Renal Pathophysiology, Instituto Nacional de Cardiología “Ignacio Chávez”, Mexico City 14080, Mexico; marthafranco@lycos.com; 5 Unidad Monterrey, Centro de Investigación y de Estudios Avanzados del Instituto Politécnico Nacional, Vía del Conocimiento 201, PIIT, Apodaca 66600, Mexico; bescalan@cinvestav.mx; 6 Department of Nephrology, Instituto Nacional de Cardiología “Ignacio Chávez”, Mexico City 14080, Mexico; adriana.mercado@cardiologia.org.mx

**Keywords:** diabetes, angiotensin II-induced hypertension, sphingomyelin, ceramide, sphingosine, shingosine-1-P, empagliflozin

## Abstract

The profile of sphingomyelin and its metabolites shows changes in the plasma, organs, and tissues of patients with cardiovascular, renal, and metabolic diseases. The objective of this study was to investigate the effect of empagliflozin on the levels of sphingomyelin and its metabolites, as well as on the activity of acid and neutral sphingomyelinase (aSMase and nSMase) and neutral ceramidase (nCDase) in the plasma, kidney, heart, and liver of streptozotocin-induced diabetic and Angiotensin II (Ang II)-induced hypertension rats. Empagliflozin treatment decreased hyperglycemia in diabetic rats whereas blood pressure remained elevated in hypertensive rats. In diabetic rats, empagliflozin treatment decreased sphingomyelin in the plasma and liver, ceramide in the heart, sphingosine-1-phosphate (S1P) in the kidney, and nCDase activity in the plasma, heart, and liver. In hypertensive rats, empagliflozin treatment decreased sphingomyelin in the plasma, kidney, and liver; S1P in the plasma and kidney; aSMase in the heart, and nCDase activity in the plasma, kidney, and heart. Our results suggest that empagliflozin downregulates the interaction of the de novo pathway and the catabolic pathway of sphingolipid metabolism in the diabetes, whereas in Ang II-dependent hypertension, it only downregulates the sphingolipid catabolic pathway.

## 1. Introduction

Numerous human studies have shown that, in cardiovascular, renal, and metabolic diseases, the profiles of sphingomyelin [1,2,3,4] and its metabolites ceramide [5,6,7,8,9,10,11], sphingosine [12], and sphingosine-1-phosphate (S1P) [13,14] are altered (reduction or elevation) in the plasma, organs (liver and heart), and tissues (skeletal muscle and adipose). Most of these studies focused on the determination of ceramide in plasma. However, it is necessary to perform preclinical studies to determine the content of sphingomyelin and its bioactive metabolites in plasma and organs such as the brain, liver, heart, and kidney, because the sphingolipid metabolism imbalance can be affected directly or indirectly in various organs. 

On the other hand, changes in the expression or activity of the enzymes that participate in sphingolipid metabolism may explain the alterations in their profile. In the catabolic pathway, sphingomyelinase (SMase) hydrolyzes sphingomyelin to release ceramide, which is hydrolyzed into sphingosine and S1P by ceramidase (CDase) and sphingosine kinase (SK), respectively [15]. Concerning the expression at the mRNA level of the enzymes involved in the synthesis (serine palmitoyltransferase) and degradation of ceramide (SMase, CDase, and SK-1), the levels of these enzymes were increased in intra-abdominal adipose tissue and the myocardium of obese patients with or without type 2 diabetes [16,17]. In another study, the level of SK expression decreased in the brain of stroke-prone spontaneously hypertensive rats and S1P receptor expression was increased in the kidney of stroke-resistant spontaneously hypertensive rats [18].

Regarding enzyme activity, secretory SMase activity increased in the serum of patients with type 2 diabetes, chronic heart failure, or acute coronary syndromes [19,20,21]. In the adipose tissue of obese non-diabetic or diabetic patients, the activity of serine palmitoyltransferase, neutral and acid CDase (nCDase and aCDase) was increased, while the aSMase activity was decreased [7]. Therefore, drugs that modify the expression or activity of the enzymes involved in sphingolipid metabolism are attractive candidates for the treatment of cardiovascular, renal, and metabolic disease.

Empagliflozin is a sodium-glucose co-transport 2 (SGLT2) inhibitor that reduces renal glucose reabsorption in type 2 diabetes patients [22]. SGLT2 inhibitors also are used for the treatment of type 1 diabetes [23], obesity [24], heart failure [25], myocardial infarction [26], atherosclerosis [27] and hypertension [28]. SGLT2 inhibitors exert cardio-renal protective effects in patients with and without diabetes, but their effects are independent of glycemic control. Interestingly, empagliflozin reduced the content of sphingomyelin and ceramide in the heart of type 2 diabetic rats [29]. In a previous study, we demonstrated that in the isolated perfused rat kidney of diabetic rats, the vasoconstriction produced by S1P increases [30]. Additionally, in the isolated perfused rat kidney, angiotensin II (Ang II) stimulates ceramide formation via the activation of nSMase [31]. Therefore, it is possible that SGLT2 inhibitors also regulate sphingolipid metabolism in the plasma and organs of diseases other than diabetes, e.g., Ang II contributes via multiple mechanisms in the development and maintenance of various pathologies including the metabolic, renal, cardiac, and hepatic [32,33]. 

The objective of this study was to investigate the effect of empagliflozin on the levels of sphingomyelin (SM) and its metabolites, as well as on the activity of aSMase, nSMase, and nCDase in the plasma, kidney, heart, and liver of streptozotocin-induced diabetic and Angiotensin II (Ang II)-induced hypertension rats. Thus, we have a model with hyperglycemia and another dependent on Ang II.

## 2. Results

### 2.1. General Characteristics of Diabetic Rats

The diabetic rats showed no weight gain, and at the end of four weeks, they presented a significant decrease in weight compared with the control. Blood glucose and urine volume were significantly higher in diabetic rats than in control rats. All of these changes decreased with empagliflozin treatment (Table 1).

### 2.2. Characteristics of Angiotensin II-Dependent Hypertension Rats

Hypertensive rats showed no weight gain and change in blood glucose concentration compared with the normotensive and were not affected by empagliflozin treatment. Ang II administration caused an increase in urine volume and blood pressure, which were not affected by empagliflozin treatment (Table 2). 

### 2.3. Sphingomyelin Content in Diabetic and Hypertensive Rats

**Figure 1:**
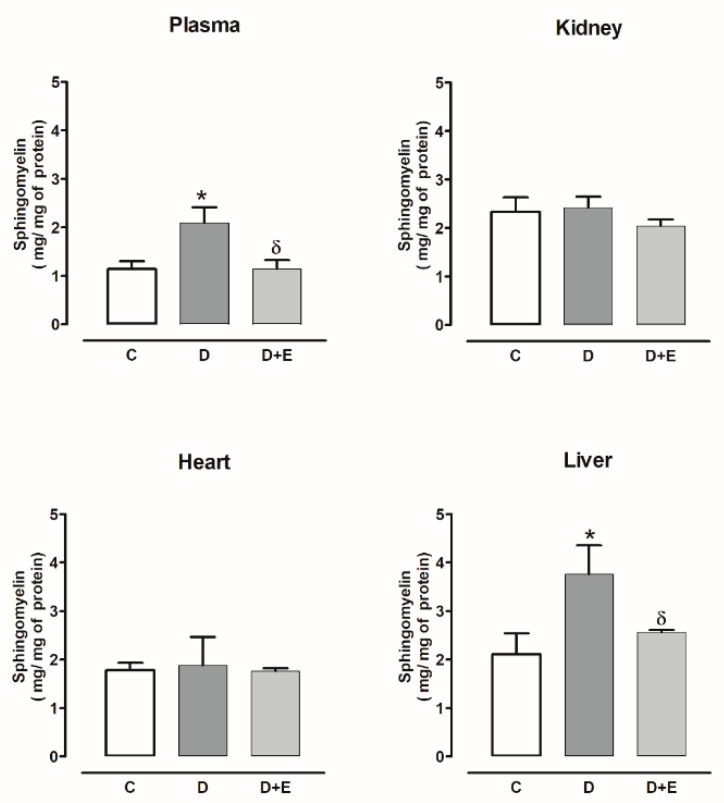
The effect of empagliflozin on sphingomyelin content in the plasma, kidney, heart, and liver of diabetic rats. Control (C), diabetic (D), and diabetic rats treated with empagliflozin (D+E). Each bar represents the mean ± SE of *n* = 10. * *p* < 0.05 when compared with control; ^δ^ *p* < 0.05 when compared with diabetic rats.

Sphingomyelin was only increased in the plasma and liver of diabetic rats compared with the control group and was reduced by empagliflozin treatment. While the kidney and heart did not show changes with or without empagliflozin compared with the control group (Figure 1). 

**Figure 2:**
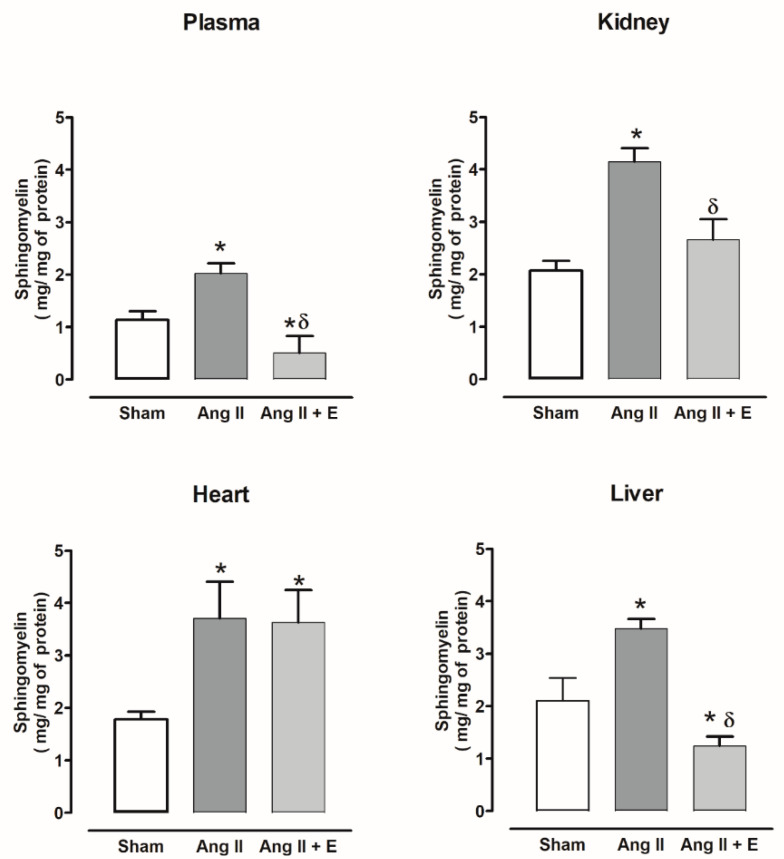
The effect of empagliflozin on sphingomyelin content in plasma, kidney, heart, and liver of hypertensive rats. Normotensive (Sham), hypertensive (Ang II-induced hypertensive rats), and empagliflozin-treated hypertensive rats (Ang II + E). Each bar represents the mean ± SE of *n* = 10. * *p* < 0.05 when compared with sham; ^δ^ *p* < 0.05 when compared with hypertensive rats.

The sphingomyelin level also increased in plasma and all the evaluated organs of hypertensive rats compared with the sham group; the empagliflozin treatment decreased sphingomyelin in the plasma, kidney, and liver, but not in the heart (Figure 2).

### 2.4. Ceramide Content in Diabetic and Hypertensive Rats

**Figure 3:**
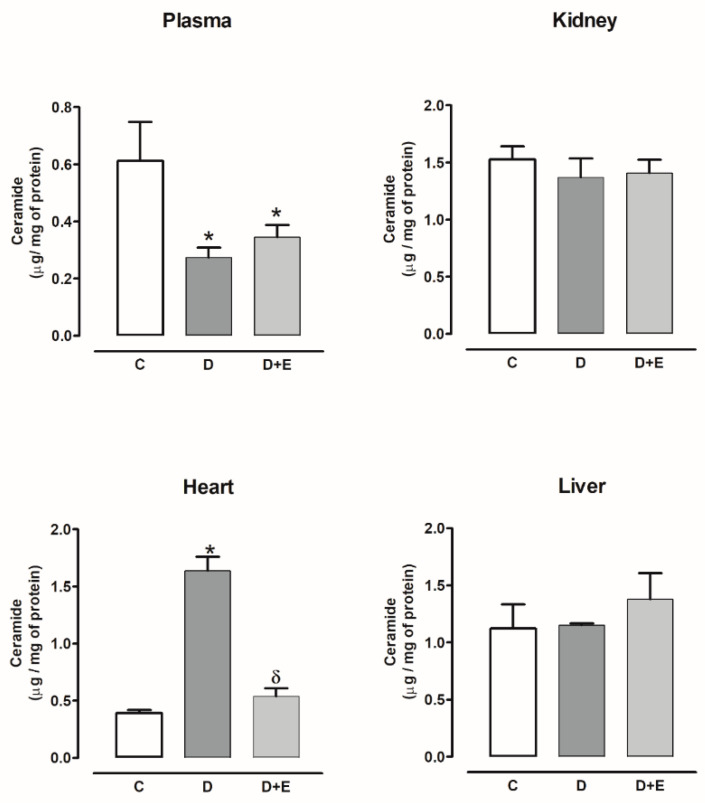
The effect of empagliflozin on ceramide content in plasma, kidney, heart, and liver of diabetic rats. Control (C), diabetic (D), and diabetic rats treated with empagliflozin (D+E). Each bar represents the mean ± SE of *n* = 10. * *p* < 0.05 when compared with control; ^&^ *p* < 0.05 when compared with diabetic rats.

The ceramide content decreased in the plasma of diabetic rats compared with the control group, and empagliflozin treatment did not modify this content, while the kidney and liver did not show changes with or without empagliflozin. The ceramide content increased in the heart compared with the control group and decreased with empagliflozin (Figure 3). 

**Figure 4:**
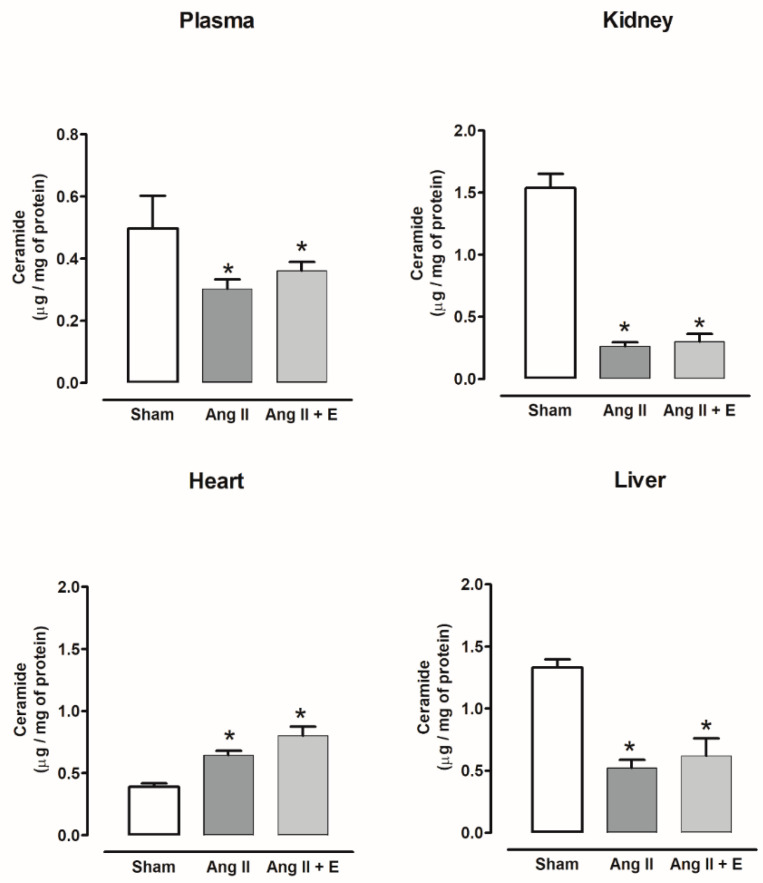
The effect of empagliflozin on ceramide content in plasma, kidney, heart, and liver of hypertensive rats. Normotensive (Sham), hypertensive (Ang II-induced hypertensive rats), and empagliflozin-treated hypertensive rats (Ang II + E). Each bar represents the mean ± SE of *n* = 10. * *p* < 0.05 when compared with sham;.

On the other hand, in plasma, kidney, and liver, the ceramide content was decreased; however, it was increased in the heart of hypertensive rats; empagliflozin treatment did not modify this content (Figure 4).

### 2.5. Sphingosine Content in Diabetic and Hypertensive Rats

**Figure 5:**
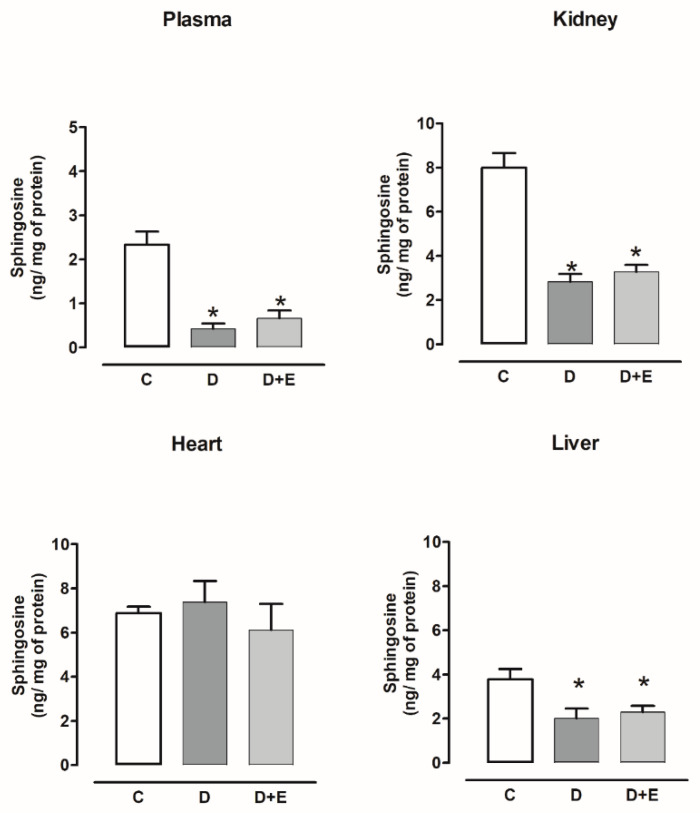
The effect of empagliflozin on sphingosine content in plasma, kidney, heart, and liver of diabetic rats. Control (C), diabetic (D), and diabetic rats treated with empagliflozin (D+E). Each bar represents the mean ± SE of *n* = 10. * *p* < 0.05 when compared with control.

The sphingosine content decreased in the plasma, kidney, and liver of diabetic rats compared with the control, and empagliflozin treatment did not modify this content. The sphingosine content did not show changes in the heart with or without empagliflozin compared with the control group (Figure 5).

**Figure 6:**
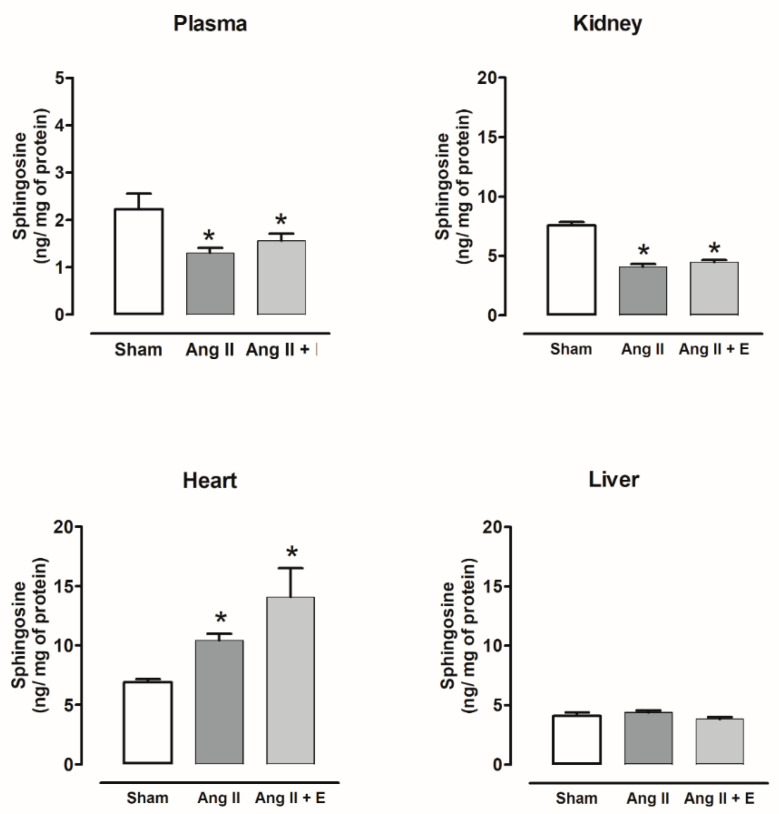
The effect of empagliflozin on sphingosine content in plasma, kidney, heart, and liver of hypertensive rats. Normotensive (Sham), hypertensive (Ang II-induced hypertensive rats), and empagliflozin-treated hypertensive rats (Ang II + E). Each bar represents the mean ± SE of *n* = 10. * *p* < 0.05 when compared with sham;.

Sphingosine content decreased in the plasma and kidney of hypertensive rats compared with the sham group, and empagliflozin treatment did not modify this content. The sphingosine content did not show changes in liver with or without empagliflozin compared with the sham group. The sphingosine content increased in the heart and empagliflozin treatment did not modify this content (Figure 6).

### 2.6. Sphingosine-1-Phosphate Content in Diabetic and Hypertensive Rats

**Figure 7:**
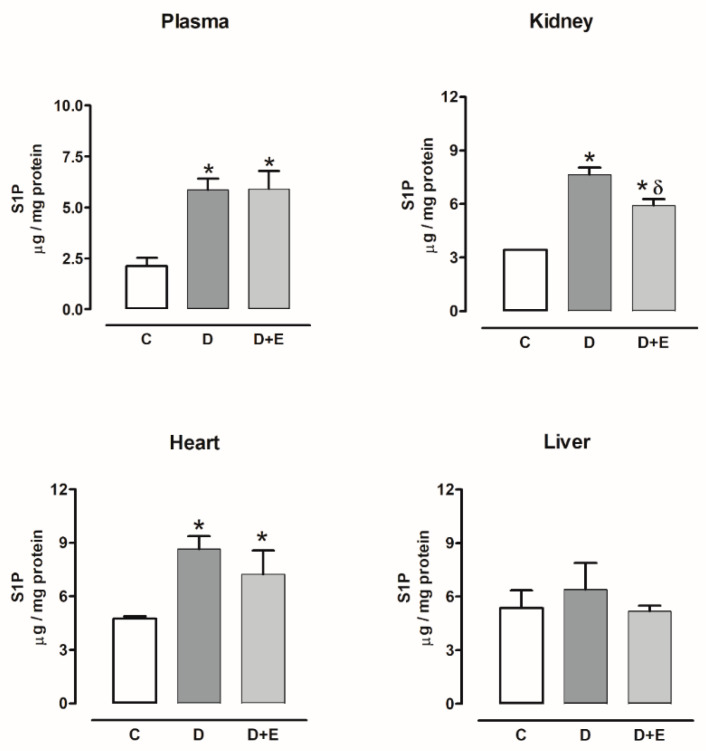
The effect of empagliflozin on sphingosine-1-phosphate content in plasma, kidney, heart, and liver of diabetic rats. Control (C), diabetic (D), and diabetic rats treated with empagliflozin (D+E). Each bar represents the mean ± SE of *n* = 10. * *p* < 0.05 when compared with control; ^δ^ *p* < 0.05 when compared with diabetic rats.

Sphingosine-1-phosphate content increased in the plasma, kidney, and heart of diabetic rats compared with the control group, and empagliflozin treatment only decreased this content in the kidney. The sphingosine-1-phosphate content did not show changes in the liver with or without empagliflozin compared with the control group (Figure 7). 

**Figure 8:**
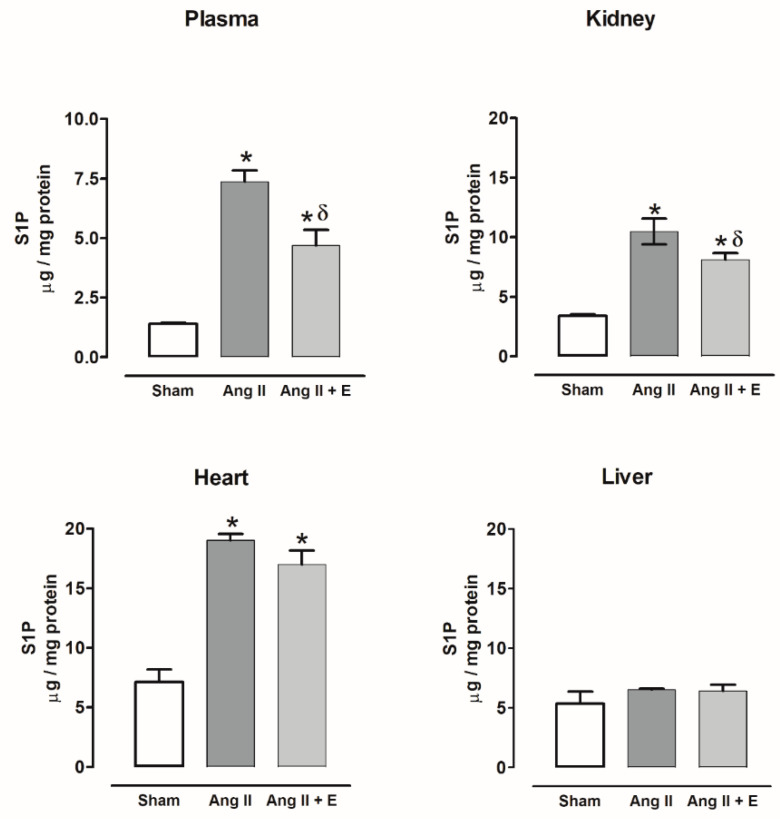
The effect of empagliflozin on sphingosine-1-phosphate content in plasma, kidney, heart, and liver of hypertensive rats. Normotensive (Sham), hypertensive (Ang II-induced hypertensive rats), and empagliflozin-treated hypertensive rats (Ang II + E). Each bar represents the mean ± SE of *n* = 10. * *p* < 0.05 when compared with sham; ^δ^ *p* < 0.05 when compared with hypertensive rats.

On the other hand, sphingosine-1-phosphate content also increased in the plasma, kidney, and heart of hypertensive rats compared with the sham group; empagliflozin treatment only decreased this content in plasma and kidney. The sphingosine-1-phosphate content did not show changes in the liver with or without empagliflozin compared with the sham group (Figure 8).

### 2.7. Sphingomielinases Activity in Diabetic and Hypertensive Rats

**Figure 9:**
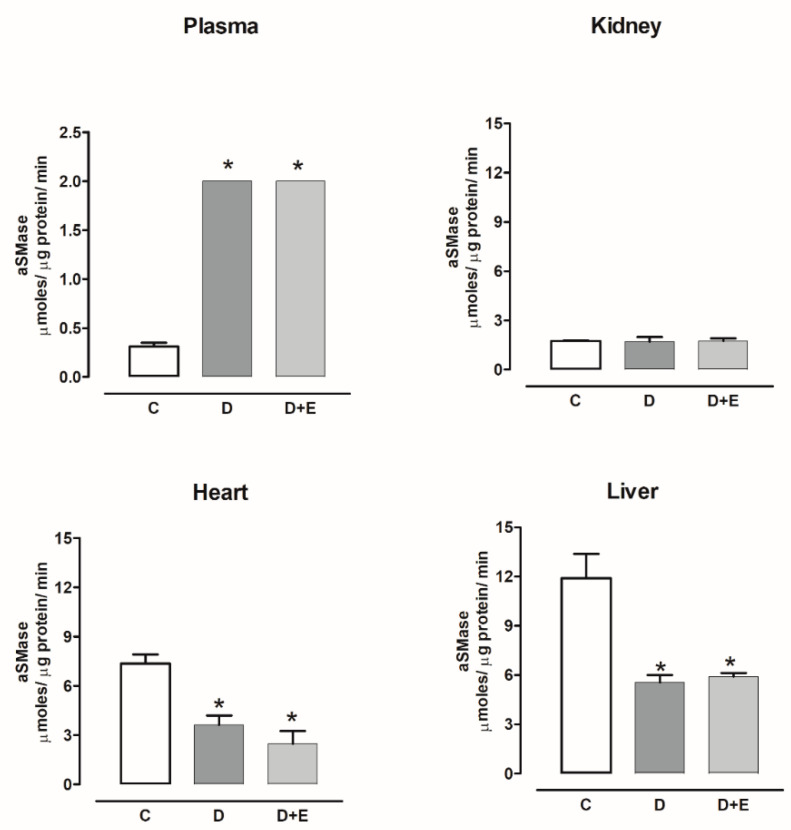
The effect of empagliflozin on acid sphingomyelinase (aSMase) activity in plasma, kidney, heart, and liver of diabetic rats. Control (C), diabetic (D), and diabetic rats treated with empagliflozin (D+E). Each bar represents the mean ± SE of *n* = 10. * *p* < 0.05 when compared with control.

The aSMase activity increased in the plasma of diabetic rats compared with the control group. The aSMase activity did not show changes in the kidney and was decreased in the heart and liver compared with the control group. Empagliflozin treatment did not modify the nSMase activity (Figure 9). 

**Figure 10:**
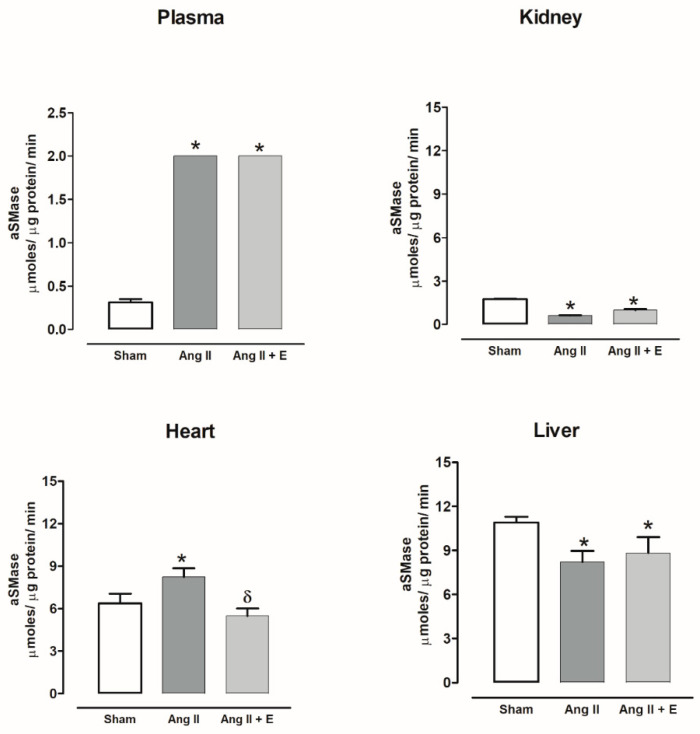
The effect of empagliflozin on acid sphingomyelinase (aSMase) activity in plasma, kidney, heart, and liver of hypertensive rats. Normotensive (Sham), hypertensive (Ang II-induced hypertensive rats), and empagliflozin-treated hypertensive rats (Ang II + E). Each bar represents the mean ± SE of *n* = 10. * *p* < 0.05 when compared with sham; ^δ^ *p* < 0.05 when compared with hypertensive rats.

The aSMase activity increased in the plasma of hypertensive rats compared with the sham group. While the aSMase activity decreased in kidney and liver, empagliflozin treatment did not modify the nSMase activity. The aSMase activity increased in the heart compared with the sham group and decreased with the empagliflozin treatment (Figure 10).

**Figure 11:**
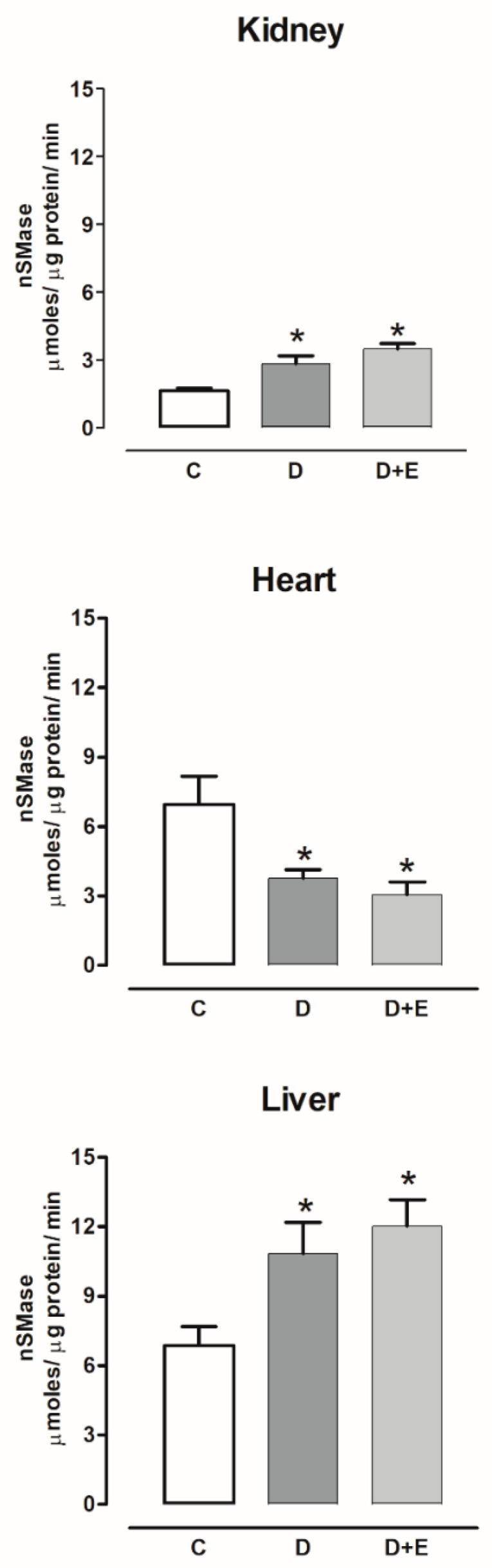
The effect of empagliflozin on neutral sphingomyelinases (nSMase) activity in kidney, heart, and liver of diabetic rats. Control (C), diabetic (D), and diabetic rats treated with empagliflozin (D+E). Each bar represents the mean ± SE of *n* = 10. * *p* < 0.05 when compared with control.

The nSMase activity increased in the kidney and liver, and decreased in the hearts of diabetic rats compared with the control group; empagliflozin treatment did not modify the nSMase activity (Figure 11).

**Figure 12:**
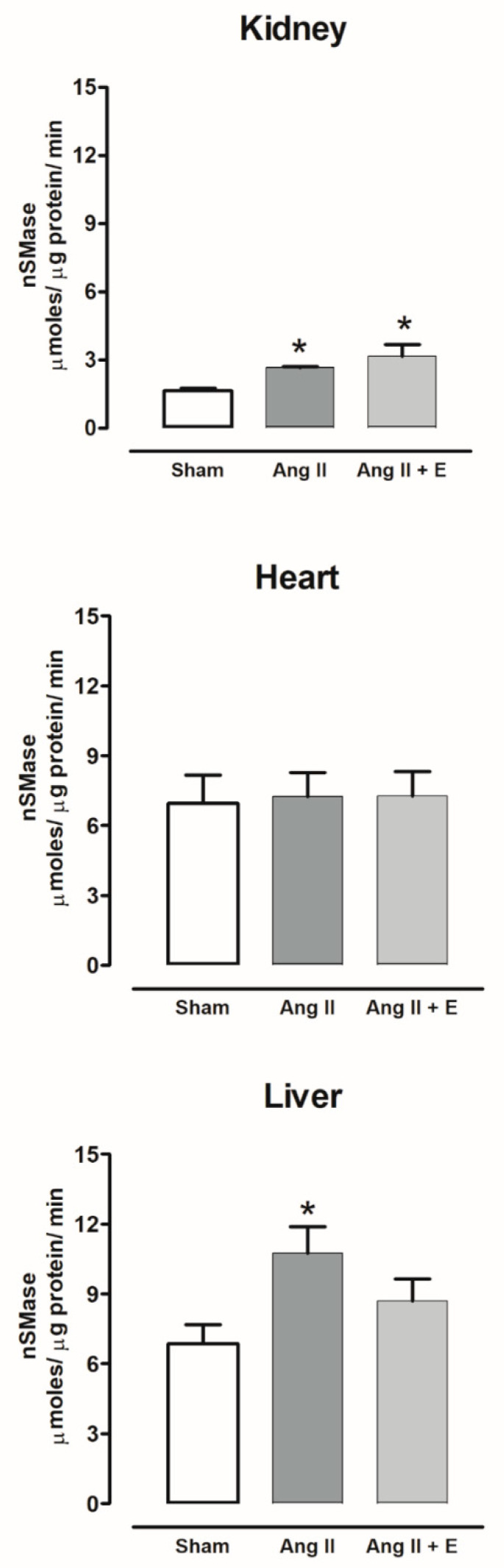
The effect of empagliflozin on neutral sphingomyelinases (nSMase) activity in kidney, heart, and liver of hypertensive rats. Normotensive (Sham), hypertensive (Ang II-induced hypertensive rats), and empagliflozin-treated hypertensive rats (Ang II + E). Each bar represents the mean ± SE of *n* = 10. * *p* < 0.05 when compared with sham.

The nSMase activity increased in the kidney and liver and was without changes in the heart of hypertensive rats compared with the sham group; empagliflozin treatment did not modify the nSMase activity (Figure 12).

### 2.8. Ceramidase Activity in Diabetic and Hypertensive Rats

**Figure 13:**
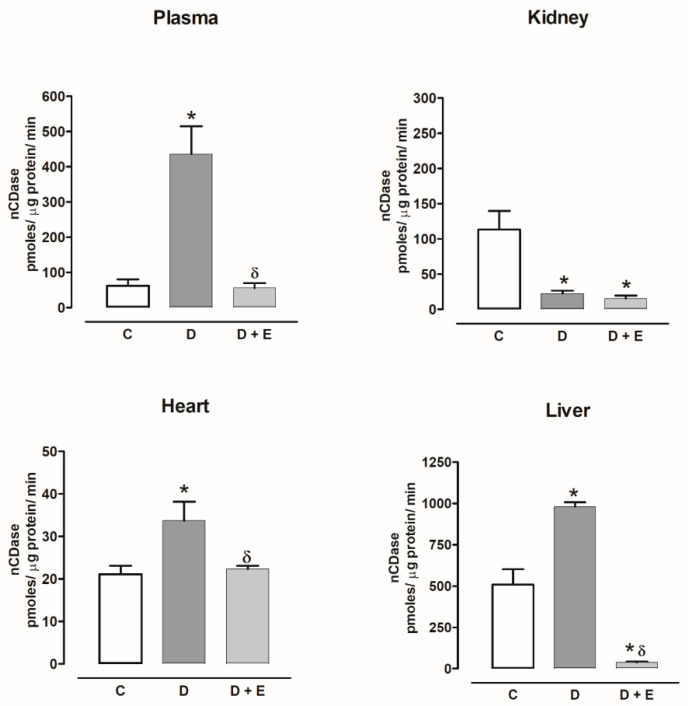
The effect of empagliflozin on neutral ceramidase (nCDase) activity in plasma, kidney, heart, and liver of diabetic rats. Control (C), diabetic (D), and diabetic rats treated with empagliflozin (D+E). Each bar represents the mean ± SE of *n* = 10. * *p* < 0.05 when compared with control; ^δ^ *p* < 0.05 when compared with diabetic rats.

The nCDase activity increased in the plasma, heart and liver of diabetic rats compared with the control group and the empagliflozin treatment decreased the activity. nCDase activity decreased in the kidney, and empagliflozin treatment did not modify the activity (Figure 13).

**Figure 14:**
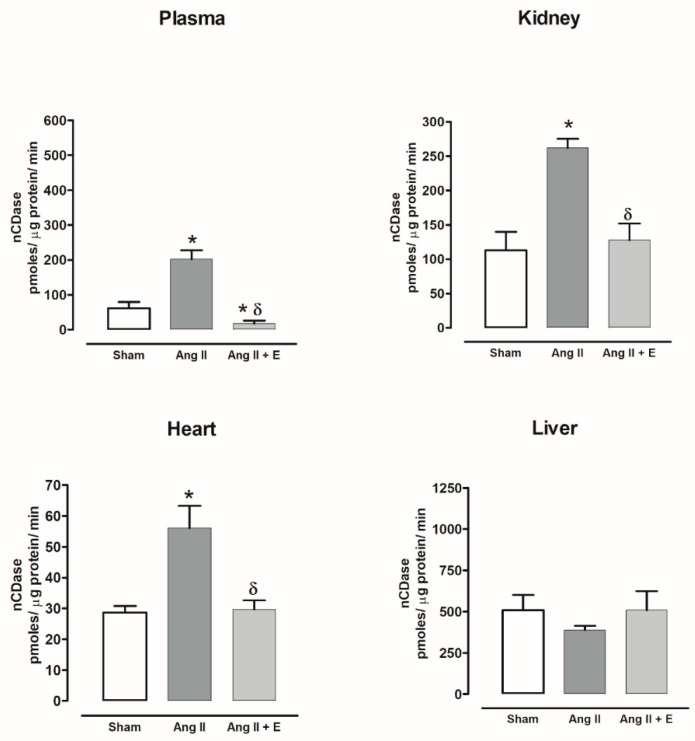
The effect of empagliflozin on neutral ceramidase (nCDase) activity in kidney, heart, and liver of hypertensive rats. Normotensive (Sham), hypertensive (Ang II-induced hypertensive rats), and empagliflozin-treated hypertensive rats (Ang II + E). Each bar represents the mean ± SE of *n* = 10. * *p* < 0.05 when compared with sham; ^δ^ *p* < 0.05 when compared with hypertensive rats.

The nCDase activity increased in the plasma, kidney, and heart of hypertensive rats compared with the sham group but decreased with empagliflozin treatment. nCDase activity in the liver did not show changes with and without treatment (Figure 14).

## 3. Discussion

In the present study, we evaluated whether empagliflozin treatment modifies the activity of the enzymes involved in sphingolipid catabolism, which can alter the contents of sphingomyelins and its metabolites in the plasma, kidney, heart, and liver of diabetic and hypertensive rats.

Sodium–glucose cotransport 2 (SGLT2) inhibitors are drugs approved for type 2 diabetes mellitus treatment that also exert cardiorenal protection in patients with and without diabetes, independent of lowering the plasma glucose concentration [34]. In this regard, in a model of type 2 diabetes, dapagliflozin decreased the renal expression of the renin–angiotensin system (RAS), oxidative stress, renal inflammation, and fibrosis [35]. 

In type 1 diabetes models, empagliflozin causes weight loss, lowers blood pressure, prevents the development of endothelial dysfunction, reduces oxidative stress and apoptosis in the pancreatic β-cells and attenuates albuminuria, renal growth, and inflammation [36,37]. Empagliflozin also prevents renal fibrosis in Ang II-dependent hypertension [38].

Interestingly, the empagliflozin treatment decreased the cardiac sphingomyelin and ceramide content in a model of type 2 diabetes [29]. In this regard, sphingomyelin, and its metabolites, such as ceramide, sphingosine, and sphingosine 1-phosphate, have been considered bioactive signaling molecules, which may directly contribute to the progression of cardiovascular, renal, and metabolic diseases [3,5,12]. However, studies are still needed to understand the mechanism or mechanisms that contribute to the damage. 

In this study, we evaluated the sphingomyelin content and its metabolites in two experimental models: diabetic and hypertensive rats. Our results show that, in the plasma and liver of diabetic rats, sphingomyelin is increased; in the heart, ceramide; and in the kidney, S1P. Moreover, increased sphingomyelin was observed in the plasma and all evaluated organs of hypertensive rats, as well as increased ceramide and sphingosine in the heart, and increased S1P in the plasma, kidney, and heart. Similar results have been reported in other experimental models, regarding the increased S1P in the plasma and heart of diabetic animals, although the liver did not show changes [39].

In the plasma of obese mice, increased sphingomyelin, ceramide, sphingosine, and S1P levels were observed, while in adipose tissue there were decreased sphingomyelin and ceramide content but increased sphingosine content [40]. In the plasma of spontaneously hypertensive rats, increased ceramide and sphingosine levels were found while in arterial tissue, only an increased ceramide content was observed [1]. 

To explain whether the changes in the sphingomyelin content and its metabolites were due to the participation of the sphingolipid catabolism enzymes, we also evaluated acid and neutral SMase activity and nCDase activity. Our results show that the aSMase activity increased in the plasma of both experimental models and only in the heart of hypertensive rats. nSMase activity increased in the kidney and liver of both experimental models. nCDase activity increased in the plasma, heart, and liver of diabetic rats, while nCDase activity increased in the plasma, kidney, and heart of hypertensive rats. 

Similar results have been reported under physiological and pathological conditions; over the course of aging, the acid and neutral SMase activity and ceramide content increased, but the sphingosine and S1P in small arteries did not change [41]. Moreover, in old age the acid and neutral SMase and nCDase activity in the brain, liver, and kidney increased [42,43,44]. The ceramide content and nSMase activity increased in the diaphragm of chronic heart failure rats [45]. In aSMase knock out mice, decreased ceramide and superoxide production was observed [46]. In the glomeruli of diabetic rats, S1P and the activity of nCDase and sphingosine kinase increased [47]. 

In addition, it is important to consider the following: in the plasma of diabetic rats, the sphingomyelin and S1P content and aSMase and nCDase activity increased, while in the liver, the sphingomyelin content and nSMase and nCDase activity increased. These results suggest that, in the liver, the sphingomyelin can be synthesized through the de novo pathway (serine palmitoyltransferase, 3-ketosphinganine reductase, ceramide synthase, dihydroceramide desaturase, and sphingomyelin synthase), which can also directly influence the sphingomyelin content in the plasma, as previously reported [48]. 

Moreover, in the liver of diabetic rats, the nCDase activity increased, however, the ceramide, sphingosine, and S1P content did not. These results suggest two possible explanations: (1) ceramide can be hydrolyzed by CDase to generate fatty acid and sphingosine, and this sphingosine can be reused through N-acylation to produce ceramide by ceramide synthase; and (2) nCDase can also catalyze the reverse hydrolysis reaction that condenses fatty acid to sphingosine, generating ceramide [49,50,51]. 

Thus, one or both pathways may contribute to ceramide formation, which can be used for sphingomyelin synthesis via sphingomyelin synthase. Interestingly, patients with type 1 diabetes also show changes in the profile of fatty acids in circulation, where they in turn overwhelm muscle, liver, heart, and other peripheral organs and tissues [52]. This may explain why we observed in the liver a decrease in the sphingosine content. In liver, empagliflozin treatment can downregulate nCDase activity to prevent the deleterious effects of sphingomyelin elevation. 

In the heart of diabetic rats, SMases activity decreased, whereas the ceramide content, nCDase activity, and S1P content increased. Our results suggest that in the heart, ceramide was not generated from the hydrolysis of sphingomyelin by the action of SMases, but can be produced by the de novo pathway. Ceramide generated by this pathway is deacylated by CDase to produce sphingosine, which can be phosphorylated by sphingosine kinases (SK1 or SK2) to S1P [15]. In this regard, our study has a limitation, in that we did not evaluate the sphingosine kinase (SK) activity.

Empagliflozin treatment decreased the ceramide content, which can be formed through the de novo pathway, and downregulated the nCDase activity. Our results agree with other studies that showed that empagliflozin treatment reduced the sphingomyelin and ceramide content, reduced the fatty acid transporter expression and decreased the expression of pro-inflammatory molecules such as interleukin-6 (IL-6), chemerin, TNF-α and MCP-1 at the mRNA level in the heart of type 2 diabetic rats [29]. Insulin treatment also decreased S1P in the heart of diabetic mice [39]. 

On the other hand, in the kidney of diabetic rats, only the nSMase activity increased, as well as the S1P content, and empagliflozin treatment decreased only the S1P content. Unexpectedly, in the heart of hypertensive rats, the content of sphingomyelin, ceramide, sphingosine, and S1P increased, as well as aSMase and nCDase activity, while in the kidney, sphingomyelin and S1P content increased, as well as the nSMase and nCDase activity. In the liver, sphingomyelin content and nSMase activity increased. 

Interestingly, in the heart of hypertensive rats, empagliflozin treatment decreased aSMase and nCDase activity, while in the kidney sphingomyelin and S1P content decreased, as well as nCDase activity, and in the liver only the content of sphingomyelin decreased. Therefore, one could speculate that, in the kidney, heart, and liver of hypertensive rats as well as in the kidney of diabetic rats, the sphingomyelin can be hydrolyzed by SMase to ceramide, and this is hydrolyzed by CDase to produce sphingosine, which can be phosphorylated by SK to S1P through the sphingolipid catabolic pathway [15]. Our results suggest that empagliflozin treatment in Ang II-induced hypertension, downregulated the sphingolipid catabolic pathway.

In this regard, it has been reported that losartan treatment in spontaneously hypertensive rats causes blood pressure and ceramide levels to decrease in the plasma and aorta [53]. This suggests that Ang II, via the AT_1_ receptor, decreases the ceramide level. In a previous study, we reported that in the isolated perfused rat kidneys, Ang II stimulates ceramide formation via the activation of nSMase [31]. 

We also previously demonstrated that, in Ang II-dependent kidney damage, the AT_1_ receptor block prevented a blood pressure increase and the production of reactive oxygen species; the empagliflozin treatment did not affect blood pressure and had a small effect on kidney damage. However, the combination of both drugs resulted in the potentiation of the effects observed with AT_1_ receptor blockade alone [54]. These findings suggest that Ang II also directly regulates sphingolipid metabolism.

It is important to note that the use of infusion pumps to increase Ang II circulation is a model that permits the fine-tuning of Ang II at set levels. Interestingly, in this model the Ang II induces the infiltration of immune cells into kidneys [55,56,57,58]. Similarly, it was shown that hyperglycemia lead to an increase in inflammation, oxidative stress and NLRP inflammasome activation [59]. 

Thus, in both experimental models the activation of immune cells can lead to an increase in the secretion of interferon γ, tumor necrosis factor α (TNF-α), and interleukin 1β (IL-1 β), IL-6, and IL-18 into circulation with potential wide-reaching effects. That is, pathological changes in an organ can instigate the release of a cascade of mediators that promote injury in other organs.

In this regard, it has been reported that IL-1 increases the aSMase activity and the concomitant activation of NF-kβ [60]. TNF-α also activates aSMase and nSMase [61]. IL-1 and TNF-α do not stimulate CDase activity [62]. 

These findings suggest that hyperglycemia and Ang II regulates directly and indirectly the sphingolipids metabolism. Therefore, if empagliflozin decreases inflammation it indirectly regulates the sphingolipids metabolism in diabetes and Ang II-induced hypertension.

## 4. Materials and Methods

### 4.1. Animal Models

#### 4.1.1. Animal Procedures

The procedures used in this study were performed in accordance with the Mexican Federal Regulation for Animal Experimentation and Care (NOM-062- ZOO-1999, published in 2001) and approved by the Institutional Committee for the Care and Use of Laboratory Animals of the Instituto Nacional de Cardiología “Ignacio Chávez”, under protocol numbers INC-CICUAL/012/2019, INC-CICUAL/005/2020, and INC-CICUAL/006/2020. 

#### 4.1.2. Streptozotocin-Induced Diabetic Rats

Male Wistar rats (300–350 g) were divided into three groups (*n* = 10 each): control, diabetic and empagliflozin-treated diabetic rats (30mg/kg/day/p. o/4 weeks). Diabetes was induced by a single intraperitoneal administration of streptozotocin (STZ) (65 mg/kg body weight) dissolved in citrate buffer (0.1 M, pH 4.5) and control rats received the same volume of citrate buffer. After 24 h of STZ administration, the blood glucose concentration was determined (Accu-check glucose monitor, Roche Diagnostics) and only rats with a blood glucose concentration greater than 300 mg/dL (18 mmol/L), empagliflozin-treatment was started and maintained for 30 days [30,63].

#### 4.1.3. Angiotensin II-Induced Hypertension Rats

Male Wistar rats (350–360 g) were divided into three groups (*n* = 10 each): normotensive (sham), hypertensive (Ang II-induced hypertensive rats), and hypertensive rats treated with empagliflozin (30 mg/kg/day/p. o/4 weeks). The rats received a two-week infusion of Ang II (Sigma, St. Louis, MO, USA) via subcutaneous osmotic minipumps (Alzet 2002; Alza, Palo Alto, CA, USA) implanted under isoflurane anesthesia. Minipumps delivered angiotensin II at a rate of 435 ng/ kg- / min; the same surgical procedure was performed but the minipump was not placed in the sham rats. After recovery from surgery, empagliflozin-treatment was started and maintained for two weeks [58].

#### 4.1.4. Sample Collection and Preparation

A total of 30 days after STZ administration, or two weeks safter the infusion of angiotensin II, the rats were placed in metabolic cages with water and food ad libitum, and urine was collected for a 24 h period and stored at −20 °C. The animals were anesthetized with pentobarbital sodium (30 mg/kg, i.p), the blood was collected in heparin tubes, and plasma was obtained by centrifugation (10,000× *g* for 5 min) and stored at −20 °C. The plasma and urine were used for the determination of glucose (Quanti Chrom^TM^ glucose assay kit).

Before dissection, organs were perfused with PBS, pH 7.4, to remove red blood cells and clots. Organs (kidney, heart, and liver) were frozen in liquid nitrogen and stored at −80 °C until use. Organs (0.1 g) were homogenized with 500 µL of Tris buffer (25 mM Tris-HCl, pH 7.4, 5 mM EDTA, 0.2% Triton X-100, and protease inhibitors) on ice. The homogenate was centrifuged at 10,000× *g* for 10 min at 4 °C. The supernatant was aliquoted and stored at −20 °C for the determination of protein, sphingomyelin, ceramide, sphingosine, sphingosine-1-phosphate, and the activity of aSMase, nSMase, and nCDase. Protein concentration was determined by Bradford assay (Bio-Rad) using BSA as a standard. 

### 4.2. Sphingomyelin Determination

For the enzymatic measurement of sphingomyelin, we used 30 μg of protein of sample which was added to individual wells of a 96-well microtiter plate that contained 100 µL of reaction buffer (50 mM Tris–HCl, pH 8 with 0.66 mM CaCl_2_) with 50 mU of *Bacillus cereus* sphingomyelinase, 1U of alkaline phosphatase, 50 mU of choline oxidase, 2U of horseradish peroxidase, 0.73 mM of DAOS (N-ethyl-N-(2-hydroxy-3-sulfopropyl)-3,5-dimethoxyaniline) and 0.73 mM of 4-aminoantipyrine. After 1 h of incubation at 37 °C, the microtiter plate was read using a microplate reader (Synergy® HTX multi-mode) at 595 nm. Standard sphingomyelin solution (5 mg/10 mL of 2% Triton X-100 ethanol solution) was used. The sphingomyelin levels were normalized to mg of protein [64].

### 4.3. Enzyme-Linked Immunosorbent Assay (ELISA) for Ceramide, Sphingosine, and Sphingosine-1-Phosphate Determination

For ceramide determination, we used 30 μg of protein sample, which was homogenized with 2 mL of chilled chloroform-methanol-1 M NaCl (1:2:0.4, *v*/*v*/*v*) and processed as previously reported [65]. Sphingosine and sphingosine-1 phosphate contents were determined using a commercial ELISA Kit, according to the manufacturer’s instruction (OKEH02615, Aviva Systems Biology, San Diego, CA, and TDS K1900, Echelon Biosciences, Salt Lake City, UT, USA, respectively) [66]. These assays were read at 450 nm (Synergy® HTX multimode). 

### 4.4. Sphingomielinase Activity

Neutral SMase (nSMase) activity was measured as previously described [31]. Fluorescence was measured using a microplate reader (Synergy® HTX multimode) at ƛ_ex_ = 545 nm and λ_em_ = 590 nm. For acid SMase (aSMase) activity, the method was performed as described for the nSMase assay, except that the reaction mixture contained 100 mM sodium acetate, pH 5 [67].

### 4.5. Ceramidase Activity

nCDase activity was measured according to previously described methods [68]. The enzymatic assay was carried out in 96-well plates; each well contained a mixture of 30 μg protein in 25 µL of a 0.2 M sucrose solution, 74.5 µL of 50 mM HEPES buffer, 1 mM CaCl_2_ at pH 7.4, and 0.5 µL of a 4 mM Rbm 14–12 substrate solution in ethanol (substrate final concentration 20 µM; ethanol final concentration 0.5%). The plate was incubated at 37 °C for 3 h without agitation. The enzymatic reaction was stopped by adding 50 µL methanol and 100 µL of sodium periodate (2.5 mg NaIO_4_/mL in 100 mM glycine/sodium hydroxide buffer at pH 10.6) was added. After incubation at 37 °C for 1 h in the dark, 100 µL /well of 100 mM glycine/sodium hydroxide buffer was added, and the fluorescence was quantified using a microplate fluorescence reader (λ_ex_ 355 nm, λ_em_ 460nm) (Synergy® HTX multimode). The same reaction mixture without enzyme was used as a blank.

### 4.6. Statistical Analysis

Values were expressed as the means ± SE. Statistical differences among groups were calculated using a one-way ANOVA, followed by the Bonferroni test, using the GraphPad Prism 6 software (GraphPad, San Diego, CA, USA); *p* < 0.05 was considered statistically significant

## 5. Conclusions

Our results suggest that empagliflozin treatment downregulates the interaction of the de novo pathway and the catabolic pathway of sphingolipid metabolism in the diabetes, whereas in Ang II-dependent hypertension, it only downregulates the sphingolipid catabolic pathway. 

## Figures and Tables

**Table 1:** General characteristics of control, diabetic and empagliflozin-treated diabetic rats.

	Control		Diabetic		Diabetic + Empagliflozin	
	Basal	Final	Basal	Final	Basal	Final
Body weight (g)	315 ± 15	330 ± 10	325 ± 8	250 ± 18 *	330 ± 10	294 ± 6.5 *
Blood glucose (mg/dL)	102 ± 8	113 ± 2.5	106 ± 4	589 ± 34 *	104 ± 6	206 ± 43 *
Urine volume (mL/24 h)	12 ± 3	12 ± 8	15 ± 2	141 ± 4.2 *	14 ± 3	50.3 ± 0.7 *

Values are mean ± EM from 10 rats. * *p* < 0.05 vs. control.

**Table 2:** General characteristics of Sham, hypertensive and empagliflozin-treated hypertensive rats.

	Sham		Ang II		Ang II + Empagliflozin	
	Basal	Final	Basal	Final	Basal	Final
Body weight (g)	324 ± 23	332 ± 20	352 ± 7	303 ± 6 *	341 ± 18	321 ± 13
Blood glucose (mg/dL)	114 ± 5	120 ± 7	116 ± 4	122 ± 11	108 ± 8	118 ± 18
Urine volume (mL/24 hrs)	12.5 ± 4	13.3 ± 9.2	14.4 ± 5.11	45 ± 8.1 *	45 ± 12	40 ± 14
Blood pressure	120 ± 5	122 ± 6.6	125 ± 4.51	195 ± 22 *	119 ± 3	195 ± 12

Values are mean ± EM from 10 rats. * *p* < 0.05 vs. Sham.

## Data Availability

Not applicable.
